# Secretory IgA and butyrate depletion in Southeast Chinese infants with cow’s milk allergy: pathogenic link and diagnostic biomarker

**DOI:** 10.1515/med-2026-1483

**Published:** 2026-09-14

**Authors:** XiaoYan Chen, XiongFeng Xie, XiaoYun Shi, XueJie Gao, Hong Ye, Rui Zhang, YouTao Chen

**Affiliations:** Fujian Children’s Hospital (Fujian Branch of Shanghai Children’s Medical Center), College of Clinical Medicine for Obstetrics & Gynecology and Pediatrics, Fujian Medical University, Fuzhou City, Fujian Province, China; Department of Gastroenterology & Nutrition Ward, Fujian Children’s Hospital, Fuzhou City, Fujian Province, China; Department of Gastroenterology, Quanzhou Maternity and Children’s Hospital, Quanzhou City, Fujian Province, China; Department of Pediatrics, Quanzhou Quangang District Hospital, Quanzhou City, Fujian Province, China

**Keywords:** cow’s milk protein allergy, secretory immunoglobulin A, butyrate, Cow’s Milk-related Symptom Score

## Abstract

**Objectives:**

To investigate the clinical significance of fecal butyrate and SIgA as non-invasive biomarkers for CMPA diagnosis.

**Methods:**

45 OFC-confirmed CMPA infants (0–24 months) and 45 healthy controls were enrolled. Fecal SIgA (ELISA/BCA) and butyrate (HPLC) were quantified. Analyses included: univariate and multivariable logistic regression (adjusting for age/weight/length), symptom correlation (Cow’s Milk-related Symptom Score, CoMiSS), and ROC-based diagnostic validation.

**Results:**

CMPA infants showed lower SIgA (6.39 ± 2.79 vs. 11.31 ± 3.32 μg/mg; p<0.001) and butyrate (298.81 vs. 810.07 μg/g; p=0.009). Both were associated with CMPA risk and inversely correlated with symptom severity. SIgA combined with CoMiSS achieved superior diagnostic accuracy (AUC=0.909) vs. CoMiSS alone (ΔAUC=0.103, p=0.010). Butyrate with CoMiSS showed no significant improvement (ΔAUC=0.002, p=0.767).

**Conclusions:**

This study indicates that low fecal SIgA and butyrate are associated with CMPA, suggesting they may play a role in the disease. The SIgA-CoMiSS integrated workflow allows non-invasive diagnosis with improved accuracy, addressing some limitations of current OFC-based protocols.

## Introduction

Cow’s milk protein allergy (CMPA), characterized by abnormal immune responses to milk-derived allergens such as α-lactalbumin, β-lactoglobulin, and casein, represents the most prevalent food allergy in early childhood. As a global public health concern, CMPA affects infant health and healthcare systems worldwide. Epidemiological studies indicate that CMPA affects approximately 2.5–3.0 % of infants globally [1], with a reported prevalence of 0.83–3.50 % among Chinese children aged 0–3 years [2]. Clinically, mild-to-moderate CMPA manifests as multisystemic symptoms, including hematochezia, diarrhea, vomiting, respiratory manifestations and cutaneous disorders. In severe cases, CMPA may lead to iron-deficiency anemia, hypoalbuminemia, growth retardation, and life-threatening anaphylaxis involving laryngeal edema or circulatory collapse. These complications affect infant health and development and impose medical and socioeconomic burdens on families and society [3]. Consequently, optimizing CMPA prevention and treatment strategies remains an important research focus.

CMPA pathogenesis involves disrupted intestinal immune tolerance. Emerging evidence indicates that butyrate, a short-chain fatty acid, may enhance immune tolerance by modulating Foxp3^+^ T regulatory (Treg) cell differentiation [4], 5], while secretory immunoglobulin A (SIgA), a core component of the mucosal barrier, may serve as a biomarker reflecting intestinal immune status [6], 7]. However, the synergistic effects of butyrate and SIgA in CMPA and their clinical value have not been clarified. By analyzing the relationship between these two factors and the risk of CMPA, symptom severity, and diagnostic efficacy, this study provides information for the immunomodulation and early diagnosis of CMPA.

## Methods

### Patient enrollment and sample collection

Infants aged 0–24 months diagnosed with cow’s milk protein allergy (CMPA) for the first time via dietary elimination and open oral food challenge (OFC) at the Department of Digestive Nutrition of Fujian Children’s hospital from June 2022 to November 2023 were enrolled in the case group. Meanwhile, 0–24-month-old non-CMPA infants undergoing routine physical examinations at the Department of Child Health were assigned to the control group. This study approved by the ethics committee of Fujian Children’s Hospital (Approval Number: No. 2022ETKLR10072, Date: October 1st, 2022). Legal guardians provided written informed consent after being informed of the study’s purpose and procedures. All procedures were performed in accordance with the ethical standards of the Institutional Review Board and The Declaration of Helsinki, and its later amendments or comparable ethical standards.

CMPA diagnostic criteria: (1) allergic symptoms after milk ingestion; (2) symptom resolution after 2–6 weeks of dietary elimination; (3) positive OFC: recurrence of original symptoms during challenge or within 2-week follow-up [3], 8].

176 infants/toddlers with suspected mild-to-moderate cow’s milk protein allergy (CMPA) were initially enrolled. Among them, 123 completed food elimination and oral food challenge (OFC) testing, while 53 withdrew due to: inability to provide stool samples (n=13), refusal to undergo OFC (n=30), concurrent respiratory/enteric infections (n=4), lost to follow-up (n=6). Of the 123 completing OFC: 45 were OFC-positive (confirmed CMPA) and 78 were OFC-negative. The 45 confirmed CMPA cases were allocated to the cases group, with 45 non-CMPA infants recruited as controls. Exclusion criteria for the control group included: use of antibiotics or probiotics within 14 days prior to enrollment, and a history of atopic dermatitis or allergic rhinitis. When recruiting the control group, we matched participants by age, sex, feeding method (breastfeeding/formula feeding/mixed feeding), and mode of delivery (vaginal delivery/cesarean section).

### General data collection

Research staff assisted legal guardians in completing structured questionnaires to systematically document demographic and medical parameters – including age, sex, weight, birth history (gestational age, delivery mode, birth measurements), feeding patterns (breastfeeding status, formula types, complementary foods), clinical manifestations (gastrointestinal, dermatological, respiratory symptoms), comorbidities, and medication records (antibiotics/probiotics usage within 14 days) for both CMPA and non-CMPA infants. Additionally, the Cow’s Milk-related Symptom Score (CoMiSS) was applied to quantify clinical symptom severity in CMPA infants, evaluating five domains: gastrointestinal disturbances (regurgitation, stool consistency changes, hematochezia), cutaneous reactions (eczema, urticaria), respiratory signs (wheezing, chronic cough), behavioral markers (prolonged crying), and systemic manifestations (growth impairment) [9].

### Fecal SIgA quantification

For the case group, specimens were obtained from infants at initial suspected CMPA diagnosis; confirmed CMPA cases were subsequently selected for SIgA analysis. Control group specimens were collected from non-CMPA infants during routine health checks using identical protocols. Fresh stools were cryopreserved at −80 °C after multisite sampling to prevent bias. Homogenized samples (0.1 g feces + 1 mL PBS) were centrifuged (3,000 rpm, 10 min, 4 °C). Fecal SIgA concentrations were measured using a commercial enzyme-linked immunosorbent assay (ELISA) kit (Human Secretory IgA ELISA Kit). Procedure: Dilute the fecal supernatant appropriately and add it to a microplate pre-coated with antibody; incubate at room temperature for 2 h. Wash the plate, add the enzyme-labeled secondary antibody, and incubate for 1 h. Wash the plate again, add the color development substrate, and incubate in the dark for 15 min. Stop the reaction with the stopping solution and read the absorbance at a wavelength of 450 nm. Total protein concentration is determined using the BCA method: Mix 25 μL of sample or standard (bovine serum albumin) with 200 μL of BCA working solution, incubate at 37 °C for 30 min, and measure absorbance at 562 nm. Calculate the standardized SIgA content (μg/mg protein) to assess the level of intestinal immunity.

### Fecal butyrate HPLC analysis

Homogenized fecal samples (0.1 g in 1 mL H_2_O) underwent overnight extraction, ultrasonication (40 kHz, 60 min), and centrifugation (10,000 × g, 10 min). Supernatants were analyzed via HPLC (Rigol L3000; Sepax HP-C18 column, 250 × 4.6 mm, 5 μm) with mobile phase (0.03 g NaH_2_PO_3_/425 mL H_2_O + 75 mL methanol + 0.2 mL 85 % H_3_PO_4_). Parameters: 0.8 mL/min flow, 30 °C, 214 nm detection, 10 μL injection. Butyrate concentrations were quantified against a standard calibration curve. Data represent intestinal butyrate levels (μg/g wet weight). Fecal butyrate concentrations underwent log_2_ transformation prior to regression analysis to meet normality assumptions. A series of concentrations was prepared using a butyric acid standard (Sigma-Aldrich), and a standard calibration curve was established. The limit of detection (LOD) for this method was 0.05 μg/g, and the limit of quantification (LOQ) was 0.15 μg/g; the intra-assay precision (repeatability) RSD was <5 %, and the inter-assay precision RSD was <8 % (n=6). Transformed values were expressed as log_2_ (μg/g). Due to limitations in the storage conditions for fecal samples, the final number of samples for butyrate testing in this study was 24 from the case group and 24 matched samples from the control group (resulting in a total of 45 pairs with complete SIgA testing, i.e., 45 cases and 45 controls).

### Statistical analysis

Analyses used SPSS 27.0. Normally distributed continuous data are mean ± SD (*t*-tests); non-normal data as median (IQR) (Mann-Whitney U tests); categorical data as n (%) (χ^2^ tests). Pearson/Spearman correlations assessed associations. Binary logistic regression modeled CMPA risk by fecal SIgA/butyrate levels adjusting for confounders. Linear regression evaluated SIgA-CoMiSS relationships. ROC curves quantified diagnostic performance of SIgA, CoMiSS, and their combination. p<0.05 defined statistical significance.

## Results

### Fecal SIgA and butyrate depletion: hallmarks of mucosal barrier dysfunction in cow’s milk protein allergy

**Figure 1:**
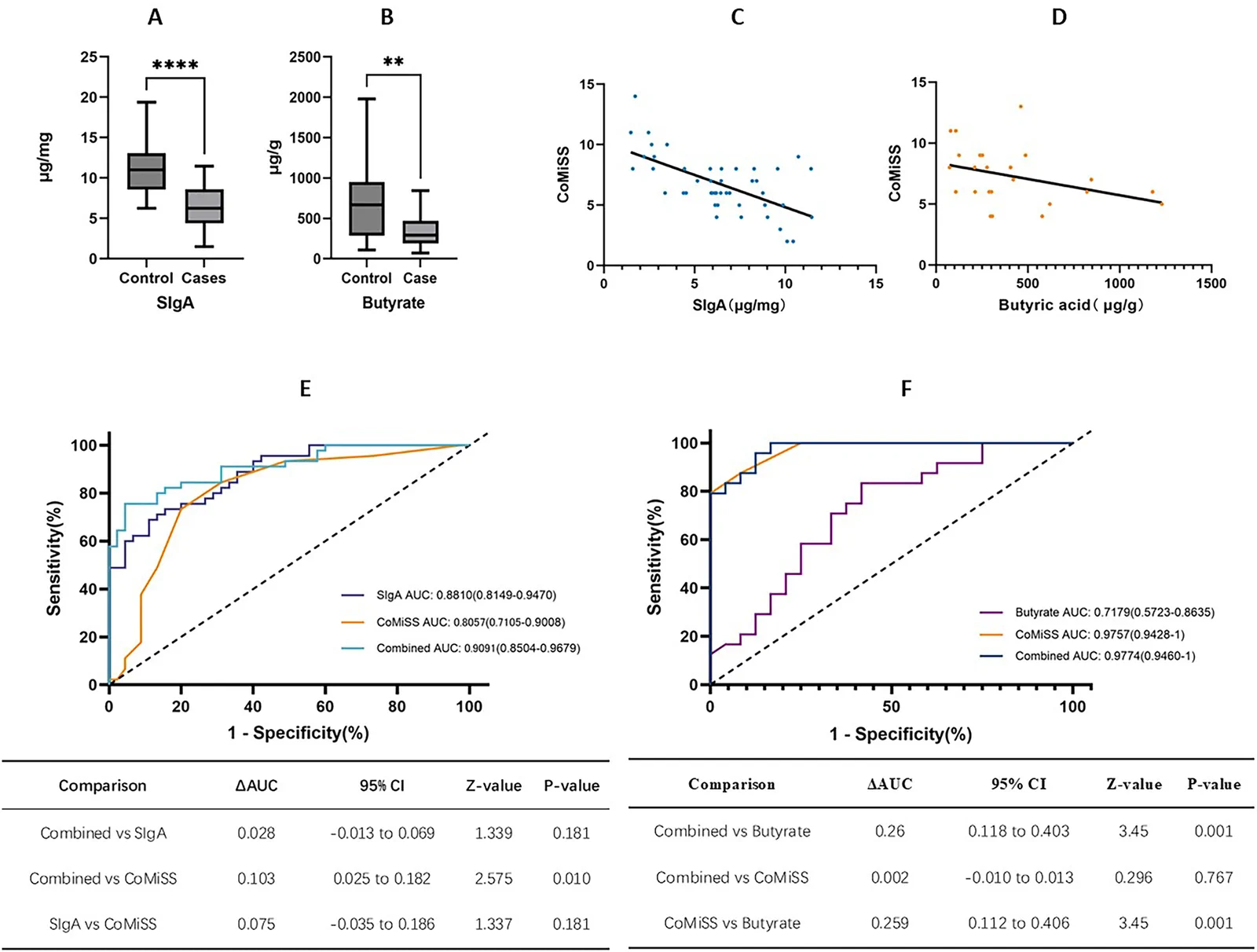
Secretory immunoglobulin A (SIgA) and butyrate levels in the feces of infants in the healthy control group and the cow’s milk protein allergy (CMPA) group. (A) Comparison of SIgA levels (number of samples tested for SIgA: 45 in the case group, 45 in the control group). (B) Comparison of butyrate concentrations (number of samples tested for butyrate: 24 in the case group, 24 in the control group). (C) Correlation between SIgA and CoMiSS scores (r=−0.621). (D) Correlation between butyrate and CoMiSS scores (r=−0.445). (E) ROC curves for the CMPA diagnostic model: ROC curves for fecal SIgA, CoMiSS, and a combined model (based on the SIgA dataset, n cases=45, n controls=45). (F) ROC curves for the CMPA diagnostic model: ROC curves for butyrate, CoMiSS, and the combined model (based on the butyrate dataset, n cases=24, n controls=24). Data are presented as mean±standard deviation (SIgA) or median [interquartile range] (butyrate).

Fecal SIgA levels were significantly lower in CMPA infants (6.39 ± 2.79 μg/mg) vs. controls (11.31 ± 3.32 μg/mg; t = 7.614, p<0.001; Figure 1A). Butyrate concentrations showed parallel reduction in CMPA infants (298.81 [IQR 344.69] μg/g) compared to controls (810.07 [IQR 1130.36] μg/g; Z=−2.618, p=0.009; Figure 1B). These results suggest differences in mucosal immunity and microbial metabolism.

### Quantification of cow’s milk protein allergy risk: fecal SIgA and butyrate deficits and multivariable regression analysis

As presented in Table 1, univariate logistic regression identified fecal SIgA (OR=0.542, 95 % CI: 0.420–0.700; p<0.001) and Log_2_-transformed butyrate (OR=0.520, 95 % CI: 0.311–0.870; p=0.013) as protective factors against CMPA. Each standard deviation decrease in SIgA increased CMPA risk by 85 % (1/0.542 ≈ 1.85), while Log_2_-transformed butyrate reduction conferred 92 % higher risk (1/0.520 ≈ 1.92).

**Table 1:** Univariate logistic regression analysis of fecal SIgA and Log2-transformed fecal butyrate as a protective factor for CMPA.

Variable	B (SE)	Wald χ^2^	p-Value	OR (95 % CI)
SIgA, μg/mg	−0.613 (0.13)	22.081	<0.001	0.542 (0.420–0.700)
Constant of SIgA	5.337 (1.165)	21.005	<0.001	207.928
Butyrate (Log_2_, μg/g)	−0.653 (0.262)	6.215	0.013	0.520 (0.311–0.870)
Constant of butyrate (log_2_)	5.799 (2.331)	6.188	0.013	330.103

SE, standard error; OR, odds ratio; CI, confidence interval. Constant term represents theoretical baseline risk when predictor=0.

After adjusting for age, weight, and recumbent length in multivariable logistic regression, both lower fecal SIgA (adjusted OR=0.465, 95 % CI: 0.327–0.662; p<0.001) and lower log2-transformed butyrate (adjusted OR=0.400, 95 % CI: 0.173–0.924; p=0.032) remained associated with increased CMPA risk (Table 2). Specifically, each 1-unit decrease in SIgA increased the odds of CMPA by approximately 115 % (1/0.465≈2.15), while a 1-unit decrease in log2(butyrate) increased the odds by 150 % (1/0.400=2.5).

**Table 2:**
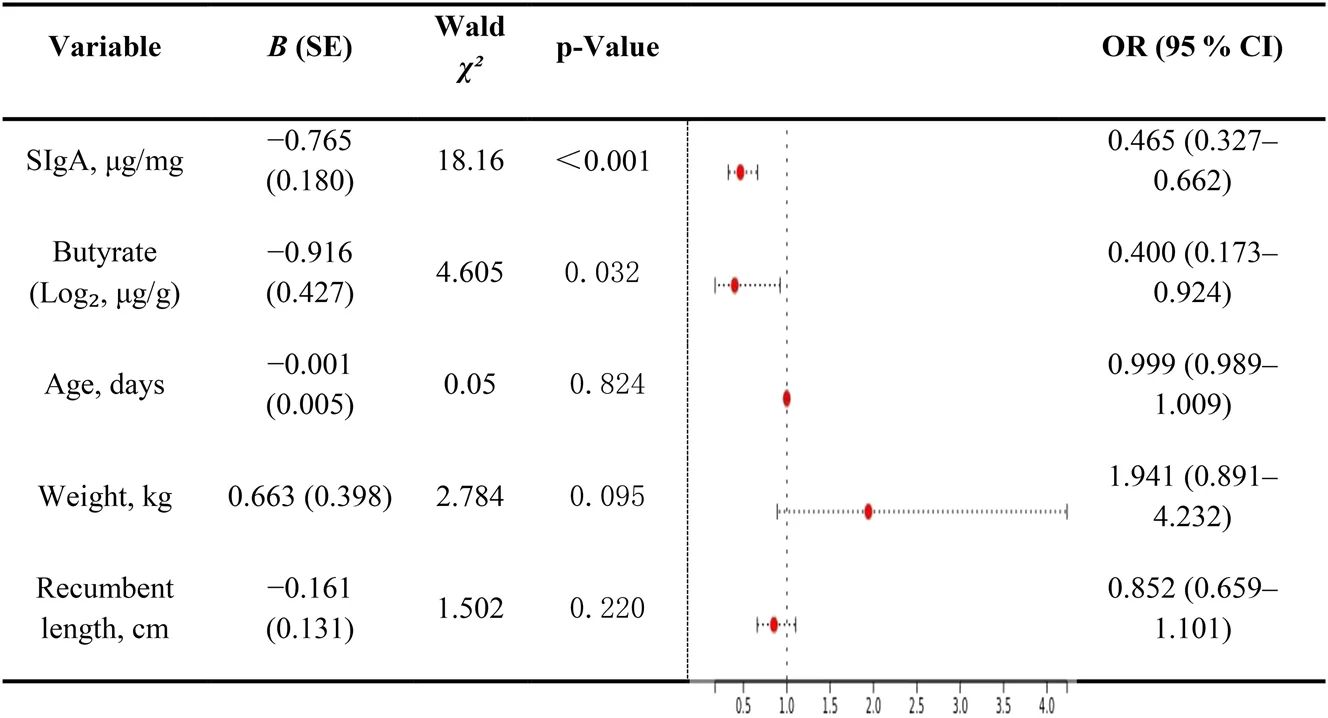
Multivariable logistic regression analysis of CMPA risk factors.

SE, standard error; OR, odds ratio; CI, confidence interval.

### Biomarker-driven symptom modulation in cow’s milk allergy: dual-phase validation of fecal SIgA and Log_2_-butyrate dose-response effects on clinical severity scores

Spearman correlation revealed significant inverse associations between CoMiSS scores and fecal biomarkers: SIgA (r=−0.621, p<0.001; Figure 1C) and Butyrate (r=−0.445, p=0.029; Figure 1D). As shown in Table 3, linear regression confirmed a robust negative association between SIgA and CoMiSS (B=−0.532, 95 % CI: −0.738 to −0.325; p<0.001), indicating that each 1 μg/mg increase in SIgA corresponded to a 0.532-point decrease in CoMiSS score. The association with log2-transformed Butyrate was also significant but less pronounced (B=−0.835, p=0.048).

**Table 3:** Linear regression analysis of association between fecal SIgA/butyrate levels and Cow’s Milk-related Symptom Score (CoMiSS).

Independent variable	Parameter	B (SE)	Standardized β	t-value	p-Value	95 % CI
SIgA, μg/mg	Slope	−0.532 (0.102)	−0.621	−5.195	<0.001	−0.738 to −0.325
	Intercept	10.153 (0.712)	–	14.255	<0.001	8.717 to 11.590
Butyrate (Log_2_, μg/g)	Slope	−0.835 (0.399)	−0.407	−2.091	0.048	−1.662 to −0.007
	Intercept	14.203 (3.335)	–	4.258	<0.001	7.286 to 21.120

SE, standard error; CI, confidence interval. Slope represents the change in CoMiSS per unit change in the independent variable. Intercept represents the estimated CoMiSS when the independent variable is zero.

### Dual-track assessment of diagnostic efficacy: synergistic enhancement by SIgA-CoMiSS integration contrasted with non-significant butyrate-CoMiSS augmentation

Diagnostic performance analysis revealed a key finding: Through ROC analysis and DeLong’s test the combination of fecal SIgA and the CoMiSS clinical score demonstrated significantly superior diagnostic accuracy for CMPA compared to CoMiSS alone (AUC=0.909 vs. 0.806; ΔAUC=0.103, 95 % CI: 0.025–0.182, p=0.010; Figure 1E). Fecal SIgA alone also showed high diagnostic value (AUC=0.881). In contrast, while Butyrate alone exhibited moderate diagnostic performance (AUC=0.763), its combination with CoMiSS did not provide significant improvement over CoMiSS alone (ΔAUC=0.002, p=0.767; Figure 1F), despite the combined model achieving a high AUC (0.976).

## Discussion

CMPA, a condition primarily manifesting in infancy and early childhood, presents diagnostic challenges in practice. Frequent errors including both misdiagnosis and underdiagnosis may precipitate long-term detrimental health outcomes. The actual prevalence of CMPA in clinical practice is <1 %, yet diagnostic rates reach 10–17 %. Optimizing CMPA diagnostic protocols is imperative. Current diagnosis relies on oral food challenge (OFC) testing, which carries operational risks and a lengthy process [10].

This study systematically analyzes the clinical significance of intestinal butyrate and SIgA in infant CMPA, providing potential biomarkers for the diagnosis of non-IgE-mediated allergies. As revealed by Li et al. [11], SIgA enhances intestinal barrier integrity, improves microbial homeostasis, and modulates Th2/Th17 immune responses – mechanisms central to cow’s milk protein allergy pathogenesis. The synergistic effect of reduced SIgA and decreased butyrate is not only associated with an increased risk of CMPA but also negatively correlated with symptom severity, suggesting an intervenable pathophysiological link – alterations in the mucosal barrier and metabolic homeostasis – which may inform future therapeutic strategies. Consistent with Kauppila et al. [12], elevated intestinal SIgA levels may serve as a key effector molecule in maintaining tolerance to milk proteins, which also suggests that increased SIgA levels may be associated with a reduced risk of CMPA. Evidence indicates that butyrate, as a bacterial metabolite, enhances immune tolerance by promoting Foxp3^+^ regulatory T cell differentiation and SIgA secretion, thereby suppressing Th2-type inflammatory responses and reducing allergy risk. Concurrently, butyrate improves the intestinal barrier through upregulation of tight junction proteins (e.g., Claudin-1, Occludin) and mucin MUC2 expression, which could limits milk protein antigen penetration and alleviates clinical symptoms [13], 14].

This study further demonstrates the diagnostic potential of SIgA. Recent studies have investigated the diagnostic utility of multiple fecal biomarkers, such as calprotectin, tumor necrosis factor-α (TNF-α), α-1-antitrypsin, β-defensin, eosinophil-derived neurotoxin, and eosinophil cationic protein [15]. While the commonly used CoMiSS provides some quantification of CMPA symptom severity, its clinician-dependent subjective nature fundamentally limits objective severity stratification [16]. This study demonstrates that fecal SIgA has high diagnostic performance: the AUC for SIgA alone was 0.881 (optimal cutoff value: 7.77 μg/mg, specificity: 88.9 %, sensitivity: 68.90 %). Based on the literature stating that “the prevalence of CMPA in Chinese children aged 0–3 years is 0.83–3.5 %,” the positive predictive value of SIgA at a cutoff value of 7.77 μg/mg is calculated to be 4.9–18.4 %, and the negative predictive value is 99.7–98.7 %. Due to the extremely low prevalence of CMPA (<5 %), the positive predictive value remains low even though the specificity of SIgA is as high as 88.9 %; conversely, the high negative predictive value suggests that a negative SIgA result can essentially rule out CMPA. In clinical practice, if a child’s fecal SIgA is <7.77 μg/mg, the likelihood of CMPA is high, and dietary avoidance or referral for an oral food challenge (OFC) can be initiated early; if the level is above this threshold and symptoms are mild, invasive testing can be deferred. This cutoff value provides an objective indicator that may help reduce unnecessary dietary restrictions or OFCs, but it requires validation in a larger sample. When combined with CoMiSS, the integrated model significantly improved diagnostic accuracy (AUC=0.897, specificity=91.1 %, Z=2.287, p=0.022 vs. CoMiSS alone). In addition, as a non-invasive biomarker, SIgA offers excellent safety and accessibility, making it particularly suitable for infants for whom an oral food challenge is contraindicated; it can also be used to monitor the effectiveness of dietary avoidance therapy over time. This approach is particularly valuable for infants contraindicated for OFC, while enabling dynamic monitoring of dietary elimination therapy efficacy.

The current study predominantly enrolled cases with gastrointestinal manifestations. Although GI symptoms represent the most common presentation of CMPA in infants, multisystem involvement (e.g., respiratory, dermatological, or mucosal symptoms) frequently occurs. Future investigations should stratify patients into clinically distinct subgroups – such as GI-dominant, skin-dominant, excessive crying–dominant, growth failure–dominant, and mixed-symptom phenotypes – with comparative assessment of fecal SIgA levels across these cohorts. This approach will determine whether SIgA levels differ significantly among CMPA subtypes and validate its diagnostic utility in non-GI-dominant CMPA (particularly cases without prominent gastrointestinal manifestations).

In this study, butyrate demonstrated lower diagnostic performance than SIgA (AUC 0.763 vs. 0.881), which may be related to the limited sample size. Furthermore, the potential therapeutic significance of butyrate in CMPA may warrant further investigation. Berni Canani et al. revealed that depletion of butyrate-producing bacteria (e.g., *Faecalibacterium prausnitzii*) and subsequent butyrate deficiency are associated with CMPA [17]. Supported by current evidence, butyrate holds greater therapeutic promise than diagnostic utility. Paparo et al. observed significantly attenuated allergic reactions in CMPA mice fed 30 mg/kg butyrate for 2 weeks compared with controls [18]. Berni Canani et al. further demonstrated that dietary interventions supplemented with butyrogenic bacteria accelerated tolerance acquisition in CMPA infants [19]. Kostadinova et al. proved that specific diets and butyrate-producing bacterial supplementation reduce CMPA incidence in murine models [20]. Therefore, the targeted delivery of butyric acid prodrugs (triglyceride nanocapsules) or the development of engineered butyric acid-producing probiotics represent promising strategies for microbiota-targeted therapy.

**Figure 2:**
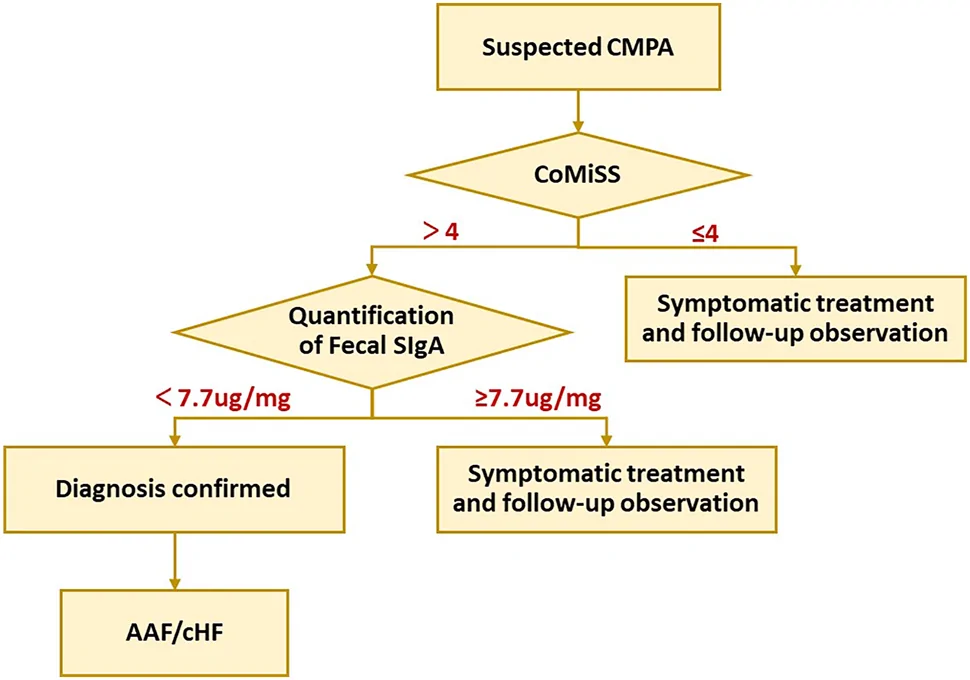
Diagnostic workflow for cow’s milk protein allergy: CoMiSS-SIgA integrated model.

Based on the results of this study, we tentatively suggest that clinicians might consider a combined SIgA+CoMiSS diagnostic workflow for CMPA: When a child presents with suspicious symptoms (elevated CoMiSS), fecal SIgA should be tested. If SIgA falls below the threshold (7.77 μg/mg), the likelihood of CMPA increases significantly. This process facilitates the earlier and more confident initiation of diagnostic avoidance diets or oral food challenge (OFC) procedures (Figure 2), or the monitoring of treatment response. However, it should be noted that this study was geographically limited to southeastern China, and the above findings require validation through multicenter studies in different populations.

Our study has several noteworthy limitations: First, the control group did not undergo an oral food challenge (OFC). Although allergies were ruled out based on clinical symptoms and medical history, there may still be a classification bias due to asymptomatic or atypical CMPA; the direction of this bias typically tends to underestimate differences between groups. Second, this study employed a cross-sectional design, which cannot establish a temporal sequence or causal relationship between fecal SIgA and butyrate levels and CMPA. We also cannot rule out the possibility of reverse causality, i.e., CMPA itself may lead to gut microbiota dysbiosis and metabolic changes, thereby causing decreased SIgA and butyrate levels. Third, the lack of longitudinal validation necessitates future prospective cohort studies that collect biological samples prior to symptom onset and conduct follow-up to clarify the temporal trajectories of biomarkers and their sequence of events relative to CMPA onset. Concurrently, the sample size should be expanded to allow for stratified subgroup analysis and better control of confounding factors such as breastfeeding and the introduction of complementary foods. We plan to conduct mechanism validation using germ-free animal models to identify therapeutic targets within the butyrate-SIgA axis that induce oral tolerance, thereby laying the theoretical foundation for treatments involving butyrate preparations or probiotics.

## Conclusions

This study provides clinical evidence of an association between intestinal butyrate and SIgA and possible involvement in immune tolerance regulation in CMPA. Low levels of both biomarkers were associated with increased allergy risk and more severe clinical symptoms, while combining SIgA with CoMiSS substantially enhanced diagnostic accuracy. Future research should focus on three areas: dynamic monitoring of butyrate/SIgA changes, strain-specific probiotic screening, and targeted interventions modulating the gut-immune axis, to advance CMPA management.
